# Preferential Consolidation of Emotional Memory During Sleep: A Meta-Analysis

**DOI:** 10.3389/fpsyg.2019.01014

**Published:** 2019-05-10

**Authors:** Gosia Lipinska, Beth Stuart, Kevin G. F. Thomas, David S. Baldwin, Elaina Bolinger

**Affiliations:** ^1^UCT Sleep Sciences and Applied Cognitive Science and Experimental Neuroscience Team (ACSENT), Department of Psychology, University of Cape Town, Cape Town, South Africa; ^2^Primary Care and Population Sciences, University of Southampton, Southampton, United Kingdom; ^3^Clinical and Experimental Sciences, Faculty of Medicine, University of Southampton, Southampton, United Kingdom; ^4^University Department of Psychiatry and Mental Health, University of Cape Town, Cape Town, South Africa; ^5^Institute of Medical Psychology and Behavioural Neurobiology, Faculty of Medicine, University of Tübingen, Tübingen, Germany

**Keywords:** consolidation, emotional memory, meta-analysis, review, sleep

## Abstract

It is uncertain whether sleep preferentially consolidates emotional over neutral material. Some studies suggest that sleep enhances emotional memory (i.e., that there are large differences in strength of memory for valenced material compared to neutral material after a sleep-filled interval, but that this difference is smaller after a wake-filled interval). Others find no such effect. We attempted to resolve this uncertainty by conducting a meta-analysis that compared valenced to neutral material after both sleep- and wake-filled delays. Standard search strategies identified 31 studies (containing 36 separate datasets) that met our inclusion criteria. Using random effects modeling, we conducted separate analyses for datasets comparing (a) negative vs. neutral material, (b) positive vs. neutral material, or (c) combined negative and positive vs. neutral material. We then specified several subgroup analyses to investigate potential moderators of the relationship between sleep and emotional memory consolidation. Results showed no overall effect for preferential sleep-dependent consolidation of emotional over neutral material. However, moderation analyses provided evidence for stronger effects when (a) studies used free recall rather than recognition outcome measures, or (b) delayed recall or recognition outcomes were controlled for initial learning. Those analyses also suggested that other methodological features (e.g., whether participants experience a full night of sleep and a regular daytime waking control condition rather than a nap and a night-time sleep deprivation control condition) and sample characteristics (e.g. all-male or not, young adult or not) should be carefully addressed in future research in this field. These findings suggest that sleep does enhance emotional memory, but that in the laboratory the effect is only observed under particular methodological conditions. The conditions we identify as being critical to consider are consistent with general theories guiding scientific understanding of memory consolidation during sleep.

Across species, sleep plays a critical role in many physiological systems, from immune to metabolic to neurobiological (Cirelli and Tononi, 2008; Rasch and Born, 2013). One strand of human neuroscientific research has focused on the active role that sleep plays in the consolidation of newly-acquired information (Diekelmann and Born, 2010). Numerous studies suggest that healthy sleep is associated with enhanced preservation of multiple types of memory, from declarative (e.g., memory for highly complex episodes) to non-declarative (e.g., conditioning of simple stimulus-response associations). Recently, there has been an increased focus within this sleep-memory literature on ways in which sleep directs preferential processing and storage of memories that may be relevant for guiding future behavior (i.e., emotional memories; van der Helm and Walker, 2011).

A number of seminal studies (e.g., Wagner et al., 2001; Hu et al., 2006; Payne et al., 2008) suggest that emotional memory is enhanced by sleep. They report that differences in strength of memory for valenced material compared to neutral material are larger after a sleep-filled delay than after a comparable wake-filled delay. A standard interpretation of this result is that, during sleep, emotional memories are preferentially consolidated so that they are relatively easily accessible for retrieval, whereas neutral memories tend to fade away.

Many studies, most focusing on emotional episodic memory, have attempted to replicate this result, investigating how preferential consolidation might work and whether it might be moderated by various study-design and individual-difference factors. Although the preferential preservation of emotional compared to neutral episodes is demonstrated in some studies (Wagner et al., 2001, 2006; Hu et al., 2006; Sterpenich et al., 2007; Payne et al., 2008; Nishida et al., 2009; Prehn-Kristensen et al., 2009, 2017; Chambers and Payne, 2014), it is not in others (Wagner et al., 2007; Atienza and Cantero, 2008; Sterpenich et al., 2009; Baran et al., 2012; Cunningham et al., 2014a; Morgenthaler et al., 2014; Tempesta et al., 2015, 2017; Cellini et al., 2016; Jones et al., 2016, 2018; Alger et al., 2018; Bolinger et al., 2018). A central problem in this literature is vast cross-study differences in methodology, and consequent difficulties in accounting for sources of discrepancy in results. These methodological variations include the timing and duration of the sleep condition, the type of waking control used, the primary outcome measure, nature of encoding encouraged by the study protocol, gender composition of the sample, and age range studied. Hence, the aggregate literature has not answered the questions of (a) whether there are large differences in strength of memory for valenced material compared to neutral material after a sleep-filled interval, but smaller differences after a wake-filled interval, and (b) if sleep does enhance emotional memory, under which experimental conditions is the effect observed.

We took a meta-analytic approach to answering these questions. We reviewed 31 studies that reported memory for emotionally valenced material compared to neutral material over any period of sleep (whole night or nap) compared to a matched period of waking or sleep deprivation (i.e., wakefulness during either the day or the night).

A series of initial analyses assessed strength of memory for (i) valenced compared to neutral material after a sleep-filled interval, and (ii) valenced compared to neutral material after a wake-filled interval. Secondary analyses examined potential moderators of the enhancement effect.

## Methods

### Study Selection

Two authors (GL and EB) searched the Academic Search Premier, MedLine, PubMed, PsycARTICLES, and PsycINFO databases using the following terms: *sleep* AND affect^*^memory; *sleep* AND emotion^*^ *memory*. The search was limited to articles published in English, and the search terms were determined a priori by the research team. The same two authors then combed the reference lists of pertinent reviews (both quantitative and narrative) for other articles that met the search criteria. This search process began in April 2018 and continued through September 2018.

Two authors (EB and GL) reviewed titles and abstracts of the articles retrieved via the above-described searches (*n* = 2,029). They selected for further evaluation only those articles that included: (1) healthy human participants, and (2) comparison of a sleep condition (e.g., a nap, or a full night's sleep) to a waking control condition (e.g., sleep deprivation, or a full day's waking activity).

We obtained a full-text copy of each article meeting these criteria (*n* = 47), and then split that set among the team for detailed review. This review ensured that the studies described in the articles met the above-listed, as well as the following three additional, criteria: (3) a basic screen (self-report was permitted) for psychiatric, medical, neurological, or other conditions with the potential to affect study outcomes, (4) at least one primary outcome measure focused on memory for emotional stimuli (e.g., recognition or free recall of valenced stimuli compared to neutral stimuli), and (5) presentation of extractable data (i.e., *M* and *SD* or *SEM*, or figures from which data could be extracted). We permitted inclusion of data from studies that featured a psychiatric comparison group only if data from healthy participants were presented separately. Similarly, we permitted inclusion of data from intervention studies (e.g., those using sleep consolidation techniques via drug or sensory cue activation) only if data from intervention-free participants were presented separately. We did, however, exclude studies (Hu et al., 2006) that grouped emotional stimuli by arousal (high, low) and matched valence within those groups. We did so because the convention in this literature is to group emotional stimuli by valence (negative, positive) rather than by arousal. We placed no restrictions on publication date, participant age range, study design, or duration of the sleep condition.

**Figure 1:**
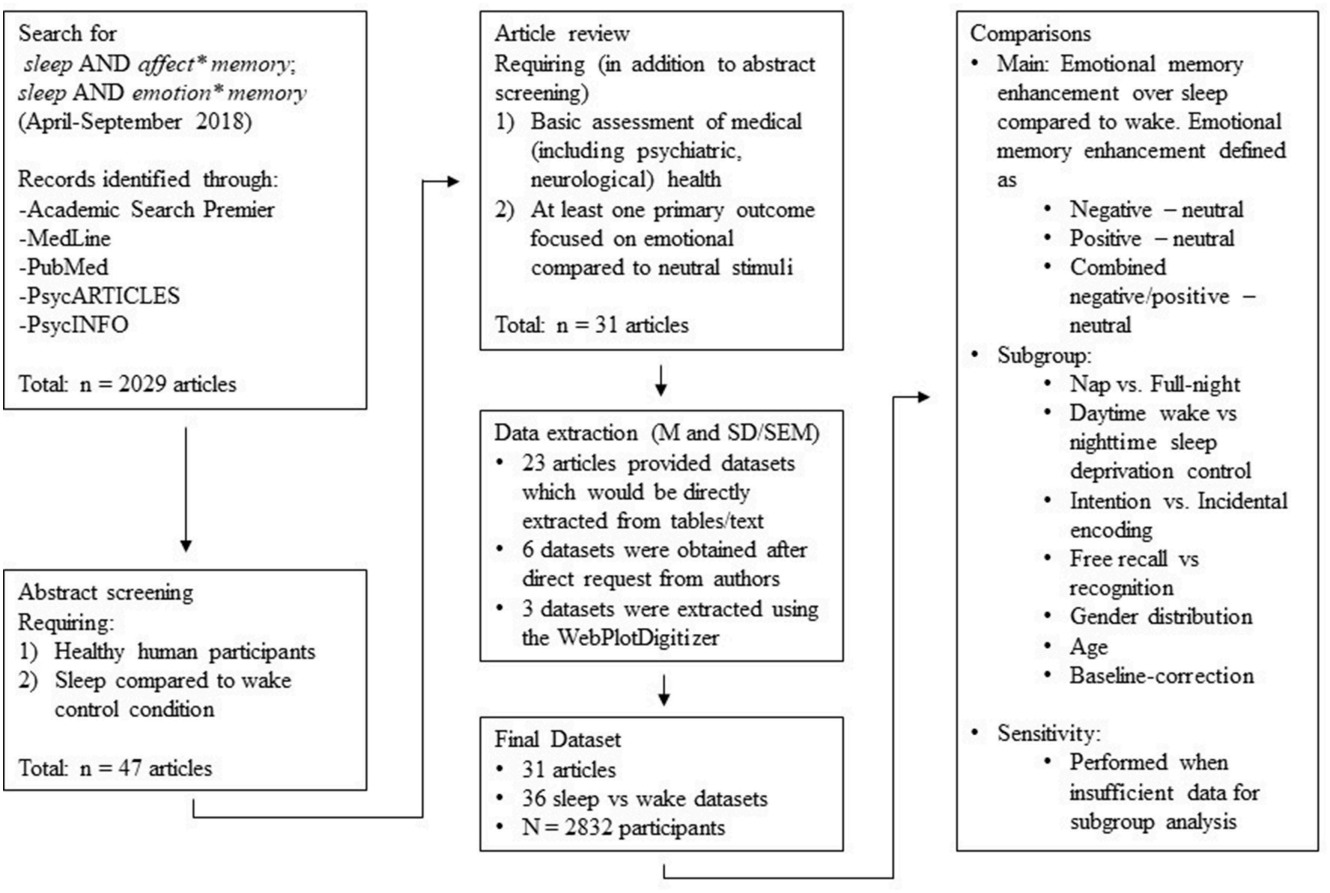
Flowchart describing the process underlying study selection.

A total of 31 articles remained in our pool after full-text screening (see Figure 1). For 23 of those, we could extract data either directly from the tables or from text contained in the paper. For the remaining 8, we requested the relevant data from the corresponding author. Five authors (representing six datasets) responded positively, but three did not. For these three, figures contained within the paper allowed us to estimate *M* and *SD* using the WebPlotDigitizer program (version 4.1; Rohatgi, 2018).

Hence, the final sample for data analysis consisted of data from 31 articles, with 36 separate datasets represented (*N* = 2,832 participants; see Table 1).

**Table 1:** Datasets included in the meta-analysis.

	**Sleep condition**		**Waking control condition**		**Sample characteristics**		**Stimulus characteristics**		
**Study/dataset**	**Type**	***n***	**Type**	***n***	**Age (years)**	**Gender**	**Type**	**Valence^a^**	**Outcome**
1. Ackermann et al., 2015	Full night	27	Full day	28	~25	Mixed	Pictures	-/+	FR^§^
2. Alger et al., 2018, dataset 1	90-min nap	15	90-min waking	15	18–39	Mixed	Foreground pictures	–	Recog. Acc.^§^
3. Alger et al., 2018, dataset 2	90-min nap	12	90-min waking	11	40–64	Mixed	Foreground pictures	–	Recog. Acc.^§^
4. Atienza and Cantero, 2008	Full night	14	Sleep deprivation	14	19–28	Mixed	Pictures	C	Recognition d′
5. Baran et al., 2012	Full night	54	Full day	28	18–30	Mixed	Pictures	–	HR
6. Bennion et al., 2015	Full night	25	Full day	17	18–34	Mixed	Pictures	–	Recog. Acc.
7. Bennion et al., 2016	120-min nap	24	120-min waking	24	18–27	Mixed	Foreground pictures	C	Recog. Acc.
8. Bolinger et al., 2018	Full night	16	Full day	16	8–11	Mixed	Pictures	–	Recog. Acc.
9. Cellini et al., 2016	90-min nap	16	90-min waking	16	20–30	Mixed	Pictures	-/+	d′
10. Chambers and Payne, 2014	Full night	15	Full day	15	~20	Mixed	Cartoon narratives	+	FR %
11. Cunningham et al., 2014a	Full night	18	Full day	21	University age	Mixed	Foreground pictures	–	Recog. Acc.
12. Cunningham et al., 2014b	Full night	21	Full day	20	University age	Mixed	Foreground pictures	–	Recog. Acc.
13. Göder et al., 2015	Full night	18	Full day	18	~28	Mixed	Pictures	–	Recog. Acc.
14. Harrington et al., 2018	Full night	14	Sleep deprivation	14	18–25	Mixed	Pictures	-/+	d′^§^^†^
15. Jones et al., 2016, dataset 1	Full night	23	Full day	19	50–80	Mixed	Pictures	–	d′
16. Jones et al., 2016, dataset 2	Full night	24	Full day	24	50–80	Mixed	Pictures	+	d′
17. Jones et al., 2016, dataset 3	Full night	52	Full day	34	18–30	Mixed	Pictures	+	d′
18. Jones et al., 2018	Full night	20	Full day	20	35–50	Mixed	Pictures	–	d′
19. Mantua et al., 2017	Full night	19	Full day	20	18–30	Mixed	Pictures	–	d′
20. Morgenthaler et al., 2014	Full night	14	Full day	14	~23	Mixed	Pictures	–	Recog. Acc.
21. Nishida et al., 2009	90-min nap	15	90-min waking	16	~23.5	Mixed	Pictures	–	d′
22. Payne et al., 2008	Full night	24	Full day	24	University age	NR	Foreground pictures	–	HR
23. Payne and Kensinger, 2011	Full night	21	Full day	21	18–29	Mixed	Foreground pictures	–	Recog. Acc.
24. Payne et al., 2015	90-min nap	23	90-min waking	34	18–26	Mixed	Foreground pictures	–	Recog. Acc.
25. Prehn-Kristensen et al., 2009	Full night	20	Full day	20	10–13	All male	Pictures	–	Recog. Acc.
26. Prehn-Kristensen et al., 2011	Full night	12	Full day	12	11–14	All male	Pictures	–	Recog. Acc.
27. Prehn-Kristensen et al., 2013, dataset 1	Full night	20	Full day	20	20–28	All male	Pictures	–	Recog. Acc.^§^
28. Prehn-Kristensen et al., 2013, dataset 2	Full night	16	Full day	16	9–12	All male	Pictures	–	Recog. Acc.^§^
29. Prehn-Kristensen et al., 2017	Full night	16	Full day	16	9–11	All male	Faces	–/+^b^	d′^§^
30. Schoch et al., 2017, dataset 1	Full night	30	Full day	28	18–35	Mixed	Pictures	C	FR %
31. Schoch et al., 2017, dataset 2	Full night	29	Full day	28	18–35	Mixed	Pictures	C	FR %
32. Sterpenich et al., 2007	Full night	21	Sleep deprivation	21	~22	Mixed	Pictures	-/+	HR^†^
33. Tempesta et al., 2015	Full night	31	Sleep-deprivation	23	~24	Mixed	Pictures	-/+	d′^§^
34. Tempesta et al., 2017	Full night	24	Sleep-deprivation	24	20–28	Mixed	Pictures from films	-/+	d′
35. Wagner et al., 2001	Late-night^c^	12	Late-night^c^	11	20–30	All male	Text	–	FR^§^
36. Wagner et al., 2007	Full night	12	Sleep deprivation	12	19–30	Mixed	Faces	–/+^d^	Recog. Acc.

Most studies used a between-subjects design. The exceptions were the following, which used a crossover design: Bolinger et al. (2018), Göder et al. (2015), Morgenthaler et al. (2014), Prehn-Kristensen et al. (2009, 2011, 2013, 2017), and Wagner et al. (2001). FR, Free Recall; Recog. Acc., Recognition Accuracy (calculated as hit rate minus false alarm rate); HR, hit rate; NR, not reported.

§ *Retention measure used*.

† *Data taken from the remember condition of a remember/know paradigm*.

a *Studies presented the following variations of valence-based analyses: -/+, negative and positive stimuli presented and analyzed separately; –, negative stimuli only; +, positive stimuli only; C, combined (i.e., negative and positive stimuli presented and collapsed together to form “emotional” variable)*.

b *Fearful (negative) and happy (positive) stimuli only; presented in separate blocks or trials*.

c *03h00–06h00*.

d *Angry (negative) and happy (positive) stimuli only; presented in separate blocks or trials*.

### Data Extraction and Coding

Three authors (GL, KGFT, and EB) extracted the following data from the 31 studies that comprised the final sample: sample size (number of participants enrolled as well as number of participants who completed the protocol), age (*M, SD*) and sex distribution of participants in each of the sleep and control conditions, study design, number and type of control conditions, type of sleep condition (nap or full night), and type of encoding (incidental or intentional). We also extracted data related to the outcome measures: type of primary outcome (recognition accuracy, as estimated by *d*', [hit rate – false alarm rate], or hit rate; and/or free recall accuracy, as estimated by the number of correctly recalled items), and the relevant statistics (*M* and *SD* for each valence category (negative, positive, neutral) within both the sleep and control conditions) for each outcome variable. Where studies reported *SE* rather than *SD*, we estimated the latter using the formula *SD* = *SE* × √*n*.

In cases where a study used more than one sleep condition, we only extracted data from the sleep condition that most closely matched the control condition (e.g., if the control was a full day's waking activity, we extracted data from the sleep condition that featured a full night's sleep, rather than from one that featured a nap). Moreover, we maintained independence of studies by using a unique control condition for each sleep condition.

We coded the extracted data into an MSExcel spreadsheet, and for each study also coded risk of bias (high, low, or uncertain) along the following dimensions: (a) clarity regarding definition of study sample; (b) clarity regarding definition and implementation of eligibility criteria; (c) clarity regarding definition of sampling strategy; (d) demographic or other matching of groups; (e) control for potential confounds (e.g., caffeine, adaptation night, daytime nap); (f) quality and validity of outcome measures; (g) percentage of participants who completed the study protocols; (h) amount of missing data; (i) adjustment of results for confounds; and (j) other.

### Risk of Bias in Included Studies

At least two members of the research team evaluated, for each study individually, features relating to sources of potential bias across 10 domains. Inter-rater disparities in evaluation were resolved by consensus.

Overall, the risk of bias analysis revealed that no studies should be considered as ‘high risk' (i.e., no study carried a high-risk profile over all or most of the bias domains). Indeed, most studies had either a low or unclear risk of bias across most domains.

#### Definition of Study Sample

All studies were judged to have a low or uncertain risk of study sample bias.

#### Stipulation of Eligibility Criteria

Many studies were judged as having an uncertain level of bias (e.g., Atienza and Cantero, 2008; Payne et al., 2008; Baran et al., 2012; Ackermann et al., 2015; Bennion et al., 2016; Alger et al., 2018). A single study was considered to have a high risk of bias (Schoch et al., 2017) as it provided no details of eligibility criteria other than describing participants as “healthy.” The remaining studies were judged to be at low or uncertain risk of bias in this domain.

#### Group Assignment and Randomization

Many studies were judged to be at low risk in this domain (e.g., Sterpenich et al., 2007; Prehn-Kristensen et al., 2013; Chambers and Payne, 2014; Harrington et al., 2018). Because details provided in other reports (e.g., Cunningham et al., 2014b; Göder et al., 2015; Jones et al., 2016; Mantua et al., 2017) were insufficient to allow clear judgements, those studies were judged to have an uncertain level of bias.

#### Matching of Study Groups

One study (Baran et al., 2012) was judged to have a high risk of bias in this domain because the gender distribution differed between the two groups and was not controlled for in the analyses. Other studies were judged to be at a low or uncertain risk of bias.

#### Methodological Attempts to Control for Potential Confounding Factors

Risk of bias was judged uncertain in four studies, due to uncertainties about (a) the location of sleep (Ackermann et al., 2015), (b) whether participants were asked not to consume alcohol- or caffeine-containing drinks prior to participation (Morgenthaler et al., 2014), or (c) whether participants were allowed naps or caffeine-containing drinks prior to participation (Payne et al., 2008; Prehn-Kristensen et al., 2013).

#### Quality and Validity of Outcome Measures

Most studies were considered at low risk of bias in this domain.

#### Participant Attrition

Two studies had high levels of participant attrition. Chambers and Payne (2014) excluded data from 46% of enrolled participants from the dataset that form part of this analysis because those individuals had prior exposure to the study materials. Morgenthaler et al. (2014) excluded data from 8 of 37 randomized participants (21.6%) from further analysis. Other studies were judged to be at low or uncertain risk of bias in this domain.

#### Other Missing Data

All studies were considered at low or uncertain risk of bias in this domain.

#### Statistical Adjustment for Potential Confounding Effects

We considered no study to be at high risk of bias in this domain. However, in four studies (Wagner et al., 2001, 2007; Atienza and Cantero, 2008; Ackermann et al., 2015) there was no explicit adjustment for the influence of potential confounding factors.

### Meta-Analytic Procedure

Due to anticipated between-study heterogeneity, we made the a priori decision to pool studies using a generic inverse variance random effects model for meta-analysis. This analysis was performed in Stata v14 with the metan command which produced the overall pooled estimate and forest plots for all comparisons. Because studies used different scales to report the same outcome measure, we have reported standardized mean differences with 95% confidence intervals. Heterogeneity is reported using the *I*^2^ statistic. The standardized effect size produced by the meta-analysis can be interpreted as per Cohen's *d* (Bradburn et al., 1998). An overall effect size of 0.2–0.5 was regarded as small, 0.5–0.8 as moderate, and more than 0.8 as large (Cohen, 1988).

We conducted separate analyses for datasets comparing (a) negative vs. neutral material, (b) positive vs. neutral material, and (c) combined negative and positive vs. neutral material. In studies reporting memory performance for both positive and negative material, we split the neutral control condition over the two comparisons to avoid unit of analysis errors (Higgins and Green, 2011).

We adopted this general analytic approach because our primary interest was the difference between memory for valenced vs. neutral stimuli. This interest is informed by the overall thrust of the literature, which claims that sleep preferentially consolidates valenced over neutral information. An alternative approach would have been to compare memory performance in response to sleep vs. waking conditions, with results from each type of stimulus computed separately. Adopting that approach, however, would mean we would not have been able to directly compare memory performance for valenced vs. neutral stimuli.

#### Subgroup Analyses

We pre-specified a number of subgroup analyses to investigate potential moderators of the relationship between sleep and emotional memory consolidation. These potential moderators included timing and duration of the sleep condition (nap vs. full-night); type of waking control condition (regular daytime waking vs. night-time sleep deprivation); type of memory encoding (intentional vs. incidental); type of outcome measure (free recall vs. recognition); gender distribution of sample (all male vs. mixed); age range of sample (young adult vs. older adult vs. children); and, finally, whether memory measures were baseline-controlled or not.

Where the chi-squared value of the test for heterogeneity indicated statistically significant differences in effect sizes between subgroups, we report effect sizes separately for each pre-defined subgroup. Where the results of the chi-squared test are not statistically significant, subgroup effects are not reported.

#### Sensitivity Analyses

Where there was insufficient data for a subgroup analysis (e.g., in cases where a subgroup contained only one study), we performed a sensitivity analysis instead and report the results excluding that study.

For studies where results were only displayed graphically, we extracted the data using the WebPlotDigitizer program and planned a sensitivity analysis excluding these studies to determine whether this method of data extraction had an influence on the results.

## Results

### Strength of Memory for Valenced vs. Neutral Material After Sleep, and After Waking

#### Negative vs. Neutral Material

**Figure 2:**
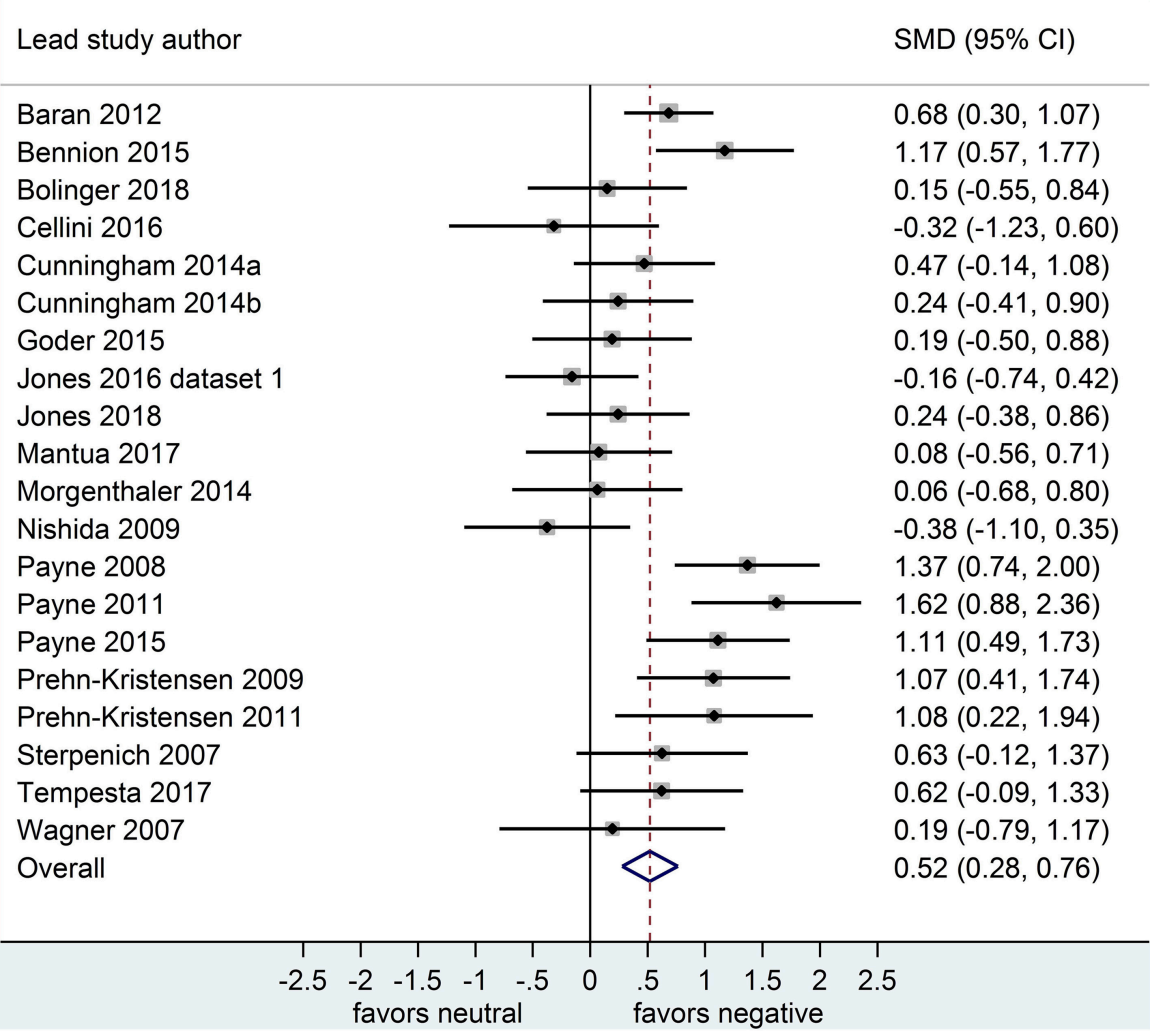
Memory for negative vs. neutral information after a sleep-filled delay (*k* = 20, *n* = 785). The *x*-axis represents effect size (Cohen's *d*). The dashed red line indicates the overall effect size, *d* = 0.52. Heterogeneity is reported as *I*^2^ = 62.1 %, *p*_Q_ < 0.001. SMD, standardized mean difference; CI, confidence interval.

**Figure 3:**
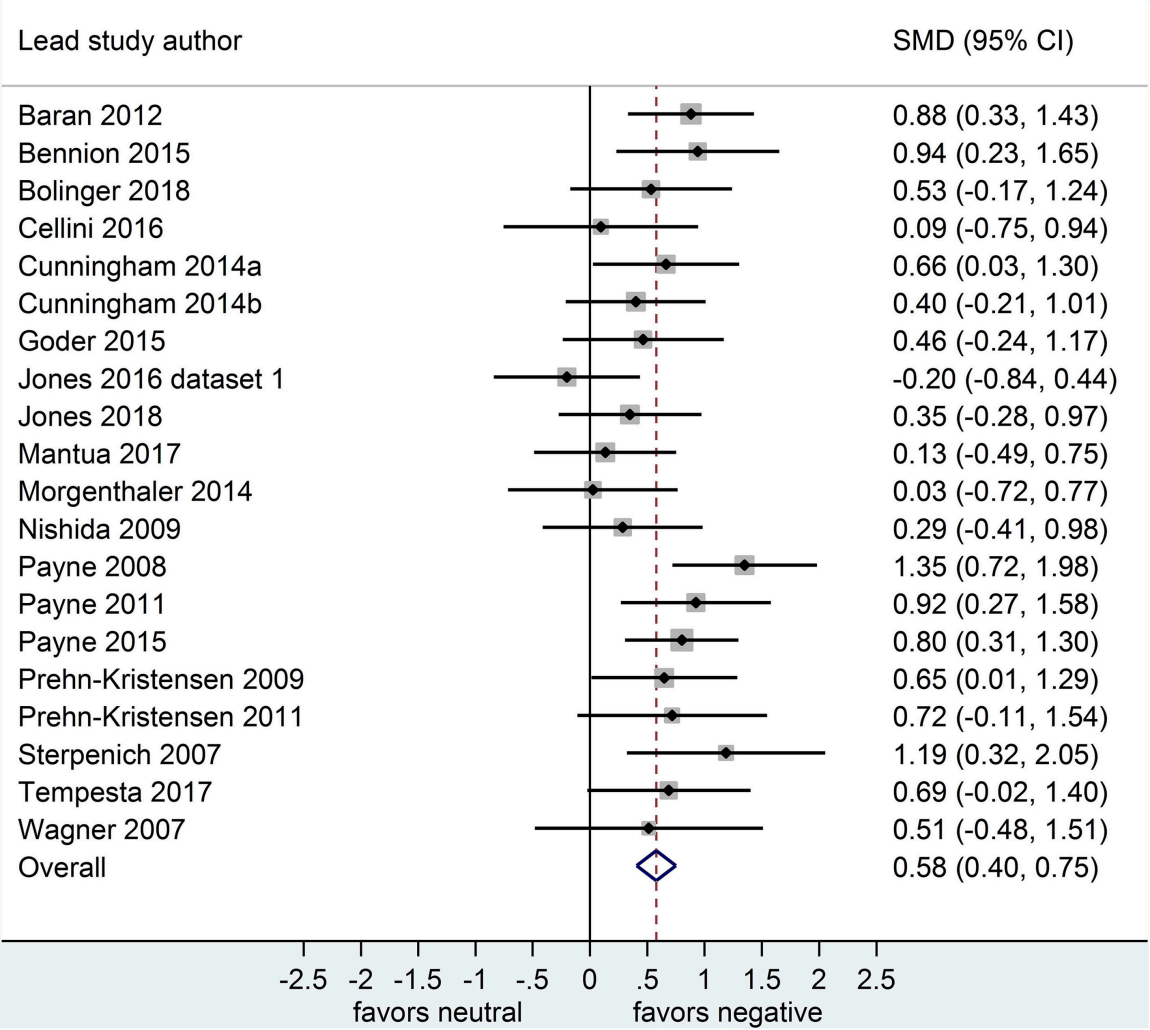
Memory for negative vs. neutral information after a wake-filled delay (*k* = 20, *n* = 739). The *x*-axis represents effect size (Cohen's *d*). The dashed red line indicates the overall effect size, *d* = 0.58. Heterogeneity is reported as *I*^2^ = 23.3 %, *p*_Q_ = 0.17. SMD, standardized mean difference; CI, confidence interval.

Separate analyses of data shown in Figure 2 (*k* = 20 datasets, *n* = 785 participants) and in Figure 3 (*k* = 20, *n* = 739) suggested that, after both sleep-filled and wake-filled delays (daytime waking and sleep-deprivation conditions, combined), participants recalled or recognized negative stimuli more readily than neutral stimuli. The effect size associated with the sleep-filled delay was similar to that associated with the waking control condition (Cohen's *d* = 0.52 and 0.58, respectively) and the confidence intervals overlapped, suggesting there is no sleep benefit for negative over neutral information.

#### Positive vs. Neutral Material

**Figure 4:**
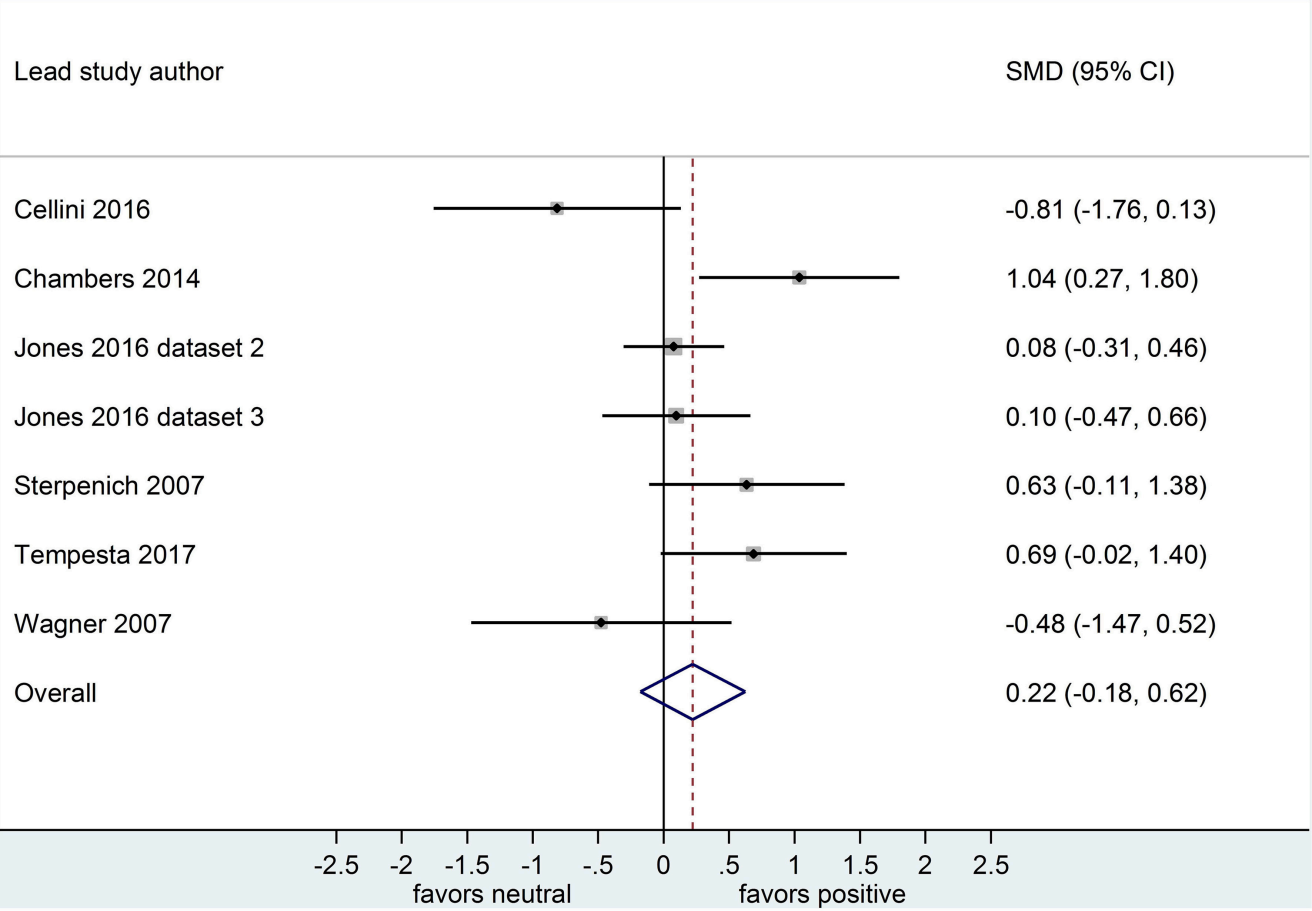
Memory for positive vs. neutral information after a sleep-filled delay (*k* = 7, *n* = 289). The *x*-axis represents effect size (Cohen's *d*). The dashed red line indicates the overall effect size, *d* = 0.22. Heterogeneity is reported as *I*^2^ = 58.3%, *p*_Q_ = 0.03. SMD, standardized mean difference; CI, confidence interval.

**Figure 5:**
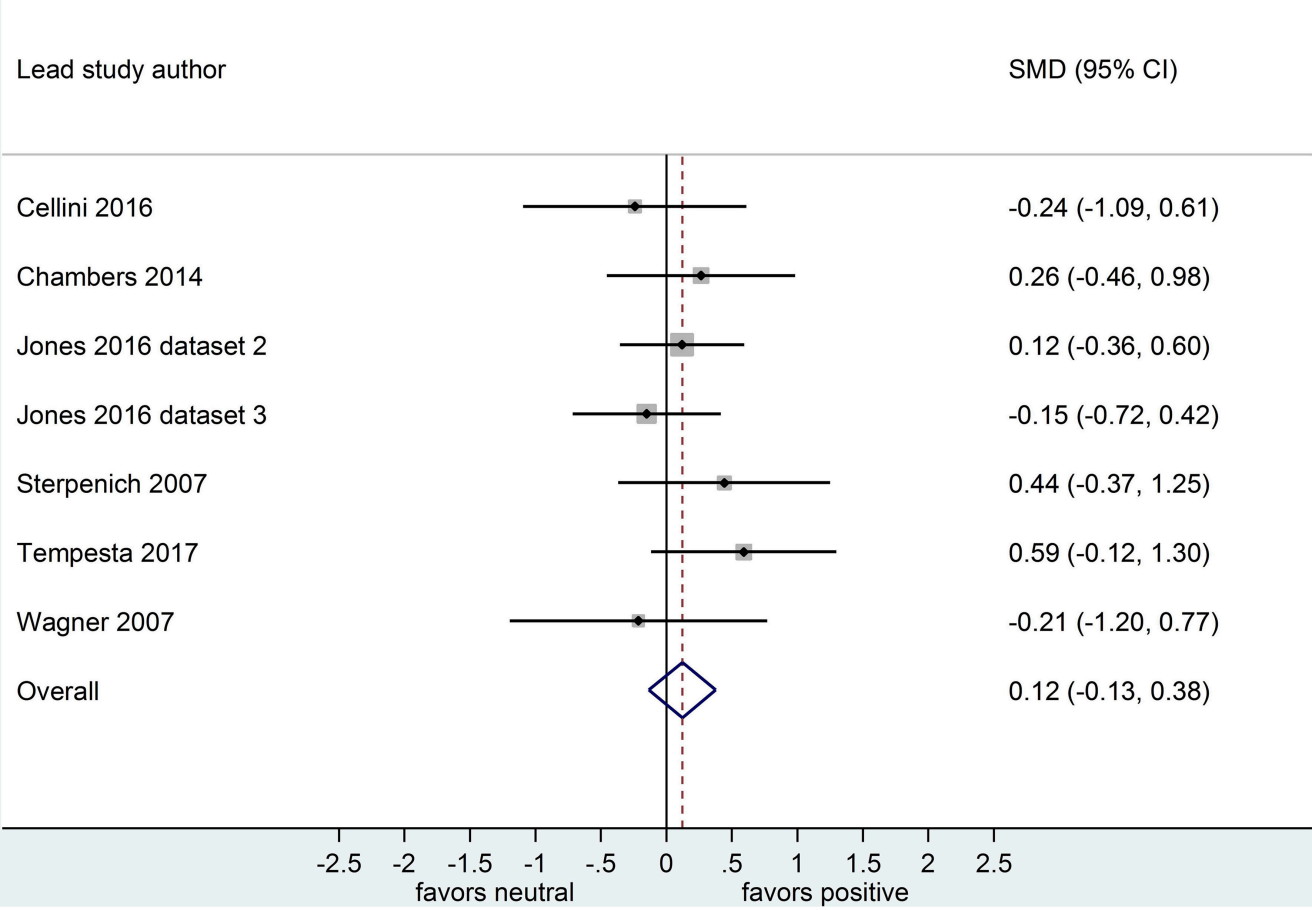
Memory for positive vs. neutral information after a wake-filled delay (*k* = 7, *n* = 251). The *x*-axis represents effect size (Cohen's *d*). The dashed red line indicates the overall effect size, *d* = 0.12. Heterogeneity is reported as *I*^2^ = 0%, *p*_Q_ = 0.62. SMD, standardized mean difference; CI, confidence interval.

Separate analyses of data shown in Figure 4 (*k* = 7, *n* = 289) and in Figure 5 (*k* = 7, *n* = 251) suggested that, after both sleep-filled and wake-filled delays (daytime waking and sleep-deprivation conditions, combined), participants did not recall or recognize positive stimuli more readily than neutral stimuli. The effect size associated with the sleep-filled delay (*d* = 0.22) was slightly larger than that associated with the waking control condition (*d* = 0.12), but in both cases the confidence intervals were quite wide and included a zero value. Moreover, the confidence intervals overlapped. Together, these data suggest there is no sleep benefit for positive over neutral information.

#### Combined Negative and Positive vs. Neutral Material

**Figure 6:**
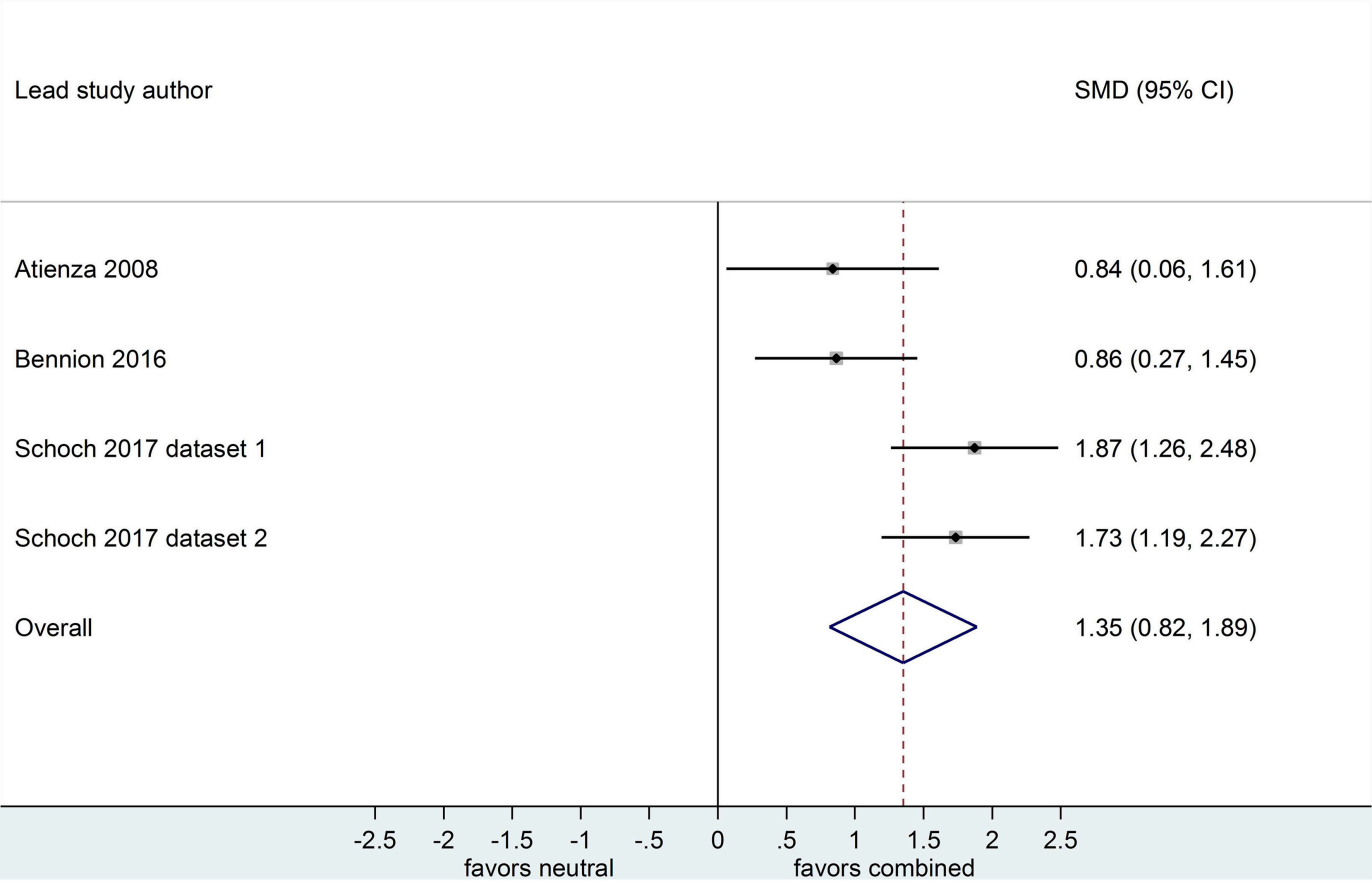
Memory for combined negative and positive vs. neutral information after a sleep-filled delay (*k* = 4, *n* = 213). The *x*-axis represents effect size (Cohen's *d*). The dashed red line indicates the overall effect size, *d* = 1.35. Heterogeneity is reported as *I*^2^ = 66.6 %, *p*_Q_ = 0.03. SMD, standardized mean difference; CI, confidence interval.

**Figure 7:**
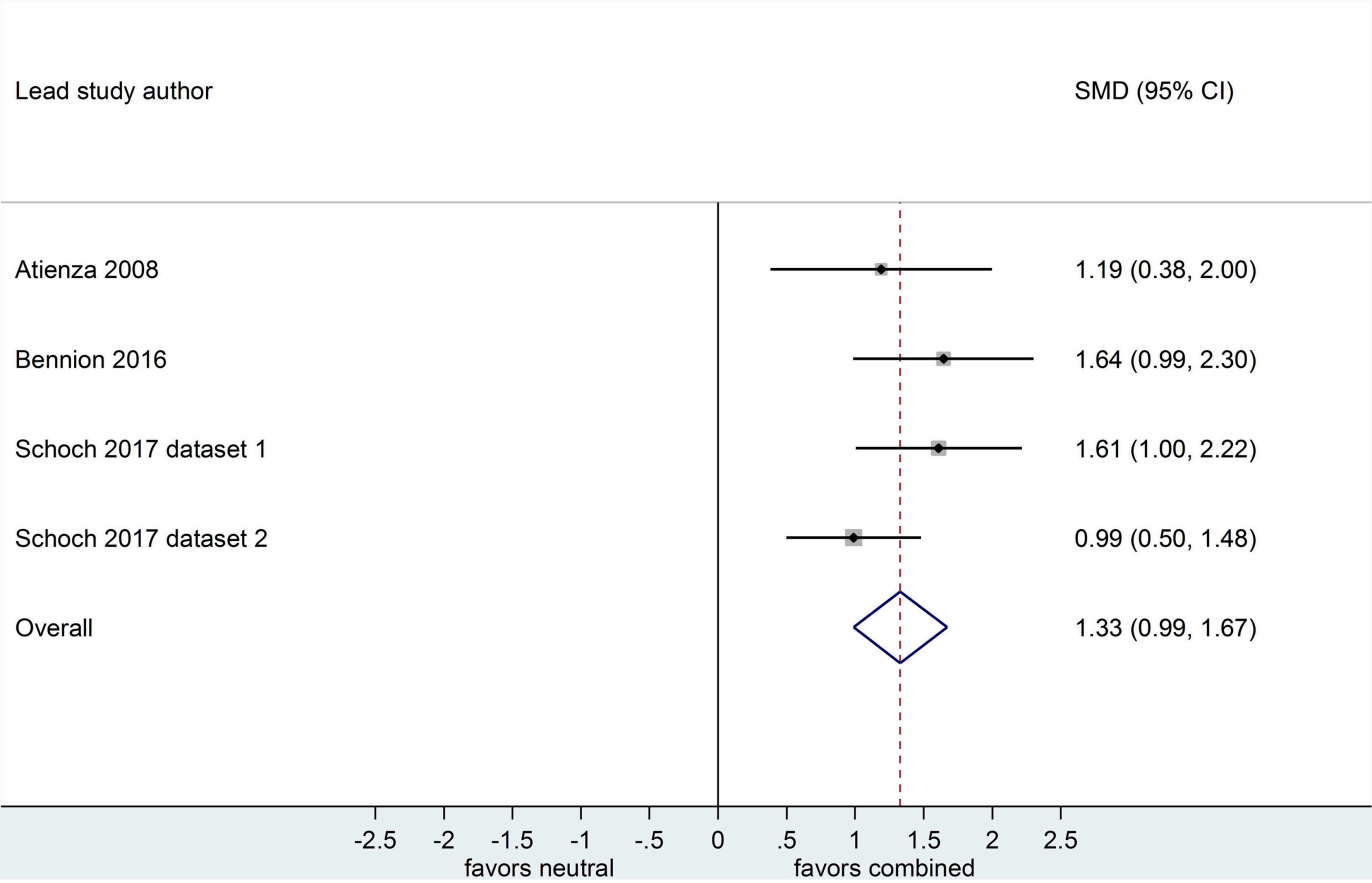
Memory performance for combined negative and positive vs. neutral information after a wake-filled delay (*k* = 4, *n* = 208). The *x*-axis represents effect size (Cohen's *d*). The dashed red line indicates the overall effect size, *d* = 1.33. Heterogeneity is reported as *I*^2^ = 17.7%, *p*_Q_ = 0.30. SMD, standardized mean difference; CI, confidence interval.

Separate analyses of data shown in Figure 6 (*k* = 4, *n* = 213) and in Figure 7 (*k* = 4, *n* = 208) suggested that, after both sleep-filled and wake-filled delays (daytime waking and sleep-deprivation conditions, combined), participants recalled or recognized valenced stimuli more readily than neutral stimuli. The effect sizes associated with the sleep- and wake-filled delays were similarly large (*d* = 1.35 and 1.33, respectively), and they overlapped, suggesting there is no sleep benefit for valenced over neutral information.

### Potential Moderators of Sleep-Enhanced Memory for Valenced Material

#### Timing and Duration of Sleep Condition

This set of analyses indicated that memory performance (emotional vs. neutral) after a sleep-filled delay might be influenced by whether participants experienced a nap or a full night of sleep.

Of the 20 datasets comparing memory performance for negative vs. neutral stimuli (see Figures 2, 3), three (Nishida et al., 2009; Payne et al., 2015; Cellini et al., 2016) made the performance comparison after either a nap or a matched waking delay, whereas 17 (*n* = 1,303) made the comparison after either a full night of sleep or a matched waking delay. Subgroup analyses detected no significant effect for either the nap vs. full-night comparison, *p* = 0.250, or for the short- vs. long-duration control comparison, *p* = 0.756.

Because only 1 nap study compared positive to neutral memory performance (Cellini et al., 2016), we undertook no subgroup analysis in this regard. Results of sensitivity analyses indicated that, after removing this study from the group, the effect size for both the post-sleep and post-waking comparisons increased slightly (see Table 2). However, the associated confidence intervals remained similar. Together, these results suggest that including or excluding the nap study from this overall pool of datasets makes little difference to the observed effect.

**Table 2:** Comparison of effect sizes: original full-sample analyses vs. sensitivity analyses.

**Potential moderator/comparison**	**Dataset removed**	**Original**		**Sensitivity**	
		***d***	**95% CI**	***d***	**95% CI**
Timing and duration of sleep condition^a^					
Positive vs. neutral	Cellini et al., 2016				
Post-sleep		0.19	−0.15, 0.54	0.28	0.03, 0.53
Post-waking		0.12	−0.12, 0.36	0.15	−0.11, 0.42
Combined negative-positive vs. neutral	Bennion et al., 2016				
Post-sleep		1.32	0.90, 1.74	1.59	1.23, 1.95
Post-waking		1.24	0.94, 1.55	1.23	0.88, 1.57
Type of waking control condition^b^					
Combined negative-positive vs. neutral	Atienza and Cantero, 2008				
Post-waking		1.24	0.94, 1.55	1.37	0.93, 1.82
Type of memory encoding^c^	Wagner et al., 2007				
Positive vs. neutral					
Post-sleep		0.22	−0.18, 0.62	0.25	0.01, 0.50
Post-waking		0.12	−0.13, 0.38	0.15	−0.12, 0.41
Combined negative-positive vs. neutral	Bennion et al., 2016				
Post-sleep		1.32	0.90, 1.74	1.59	1.23, 1.95
Post-waking		1.24	0.94, 1.55	1.23	0.88, 1.57
Type of outcome measure^d^					
Positive vs. neutral	Chambers and Payne, 2014				
Post-sleep		0.19	−0.15, 0.54	0.21	−0.13, 0.37
Post-waking		0.12	−0.12, 0.36	0.10	−0.14, 0.37
Age distribution of sample^e^					
Positive vs. neutral	Jones et al., 2016, dataset 2				
Post-sleep		0.19	−0.15, 0.54	0.21	0.13, 0.55
Post-waking		0.12	−0.12, 0.36	0.02	−0.32, 0.35
Method of data extraction^f^					
Negative vs. neutral	Atienza and Cantero, 2008; Payne et al., 2008; Bennion et al., 2016				
Post-sleep		0.55	0.31, 0.79	0.55	0.31, 0.80
Post-waking		0.55	0.38, 0.73	0.51	0.36, 0.66

Sensitivity analyses removed:

a Datasets from studies using a nap paradigm;

b Dataset from study using a sleep deprivation paradigm;

c Dataset from a study using intentional encoding during learning phase;

d Datasets from studies using free recall outcome measures;

e Dataset from a study using an older adult sample;

f *Datasets that were extracted graphically*.

Similarly, only 1 nap study compared combined negative and positive to neutral memory performance after either a sleep-filled or wake-filled delay (regular day-time waking or night-time sleep deprivation; Bennion et al., 2016). Sensitivity analyses indicated that the magnitude of the difference in effect size estimates for the two comparisons becomes larger when excluding the nap study (see Table 2). This finding suggests that the preferential consolidation of emotional over neutral material is observed more strongly after a full night of sleep than after a nap. A caveat here, though, is that the confidence intervals presented above overlap quite markedly, and hence this finding should be explored with a larger pool of datasets, particularly in the nap condition.

#### Type of Waking Control Condition

There were some suggestions from this set of analyses that memory performance (especially negative vs. neutral) after a wake-filled delay might be significantly influenced by whether participants experienced (as a control condition) regular daytime waking or night-time sleep deprivation.

Of the 20 datasets comparing memory performance for negative vs. neutral stimuli (see Figures 2, 3), three (n = 167; Sterpenich et al., 2007; Wagner et al., 2007; Tempesta et al., 2017) made the performance comparison after a period of night-time sleep deprivation *d* = 0.80, 95% CI [0.32, 1.28]. The other 17 (*n* = 1,357) made the comparison after a regular daytime waking delay, *d* = 0.56, 95% CI [0.37, 0.76]. Subgroup analysis detected a significant effect, *p* = 0.03, suggesting that although in both control conditions negative material is remembered more readily than neutral material, this effect is more pronounced in studies using sleep deprivation paradigms.

Of the 7 datasets comparing memory performance for positive vs. neutral stimuli (see Figures 4, 5), three (*n* = 54; Sterpenich et al., 2007; Wagner et al., 2007; Tempesta et al., 2017) made the performance comparison after a period of night-time sleep deprivation, *d* = 0.36, 95% CI [−0.11, 0.83]. The other four (*n* = 89) made the performance comparison after regular daytime waking delay, *d* = 0.02, 95% CI [−0.28, 0.33]. Subgroup analysis detected a significant effect, *p* < 0.001. Of note here, however, is that both confidence intervals are relatively wide, and both cross the zero midline. Hence, one might draw two conclusions: (a) this effect is more pronounced in studies using sleep deprivation paradigms, and (b) this is an unreliable finding that requires exploration in larger samples of studies.

Because only one sleep deprivation study compared combined negative and positive to neutral memory performance (Atienza and Cantero, 2008), we undertook no subgroup analysis in that regard. However, sensitivity analyses indicated that including or excluding the sleep deprivation study from this overall pool of datasets made little difference to the observed effect (see Table 2).

#### Type of Memory Encoding

This set of analyses indicated that memory for emotional vs. neutral material after a sleep- or wake-filled delay was not significantly influenced by whether participants' pre-delay encoding of the to-be-remembered material was intentional or incidental.

**Figure 8:**
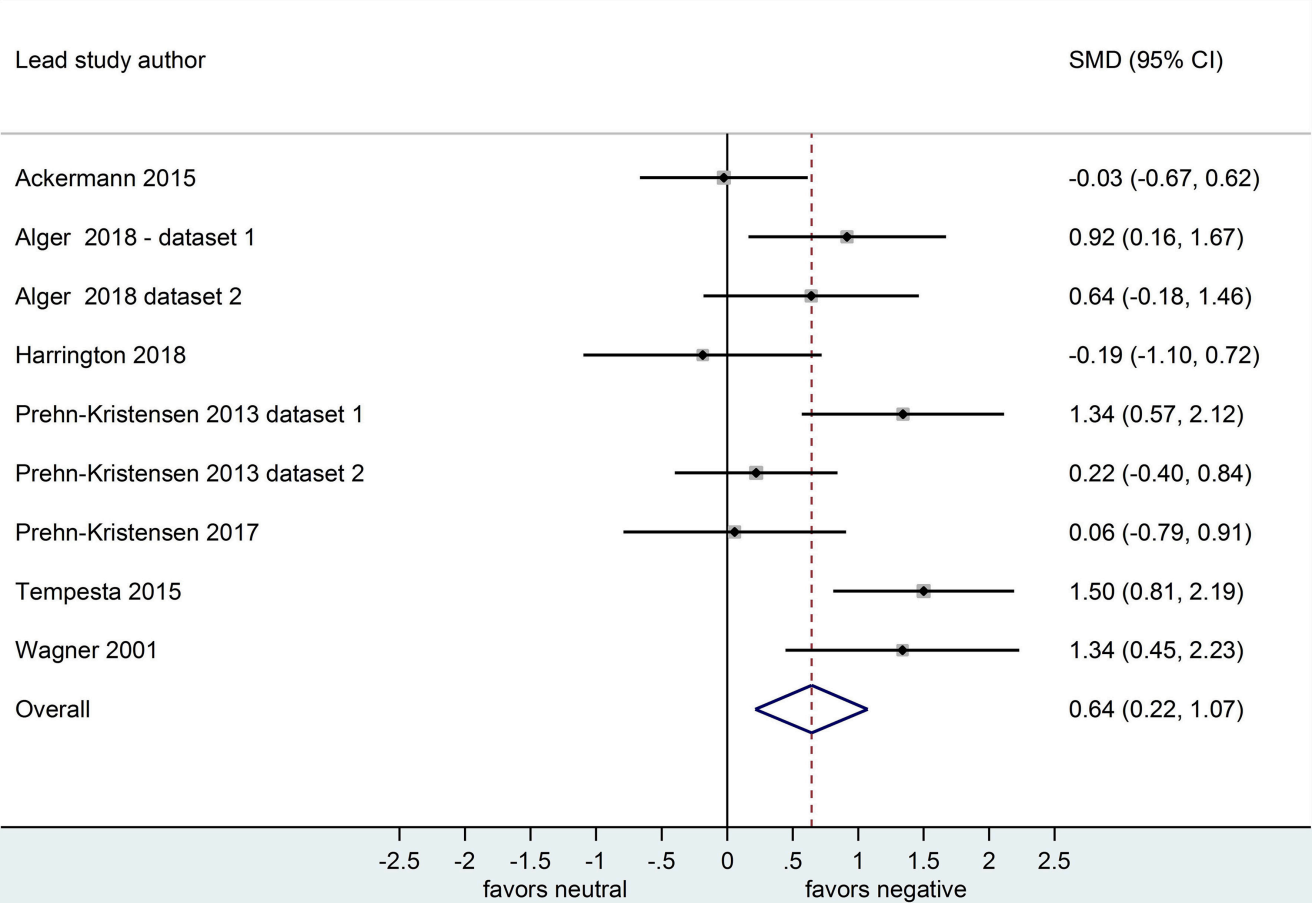
Memory performance for negative vs. neutral information after a sleep-filled delay in datasets using retention memory outcome measures (*k* = 9, *n* = 297). The *x*-axis represents effect size (Cohen's *d*). The dashed red line indicates the overall effect size, *d* = 0.64. Heterogeneity is reported as *I*^2^ = 65.0%, *p*_Q_ < 0.01. SMD, standardized mean difference; CI, confidence interval.

Of the 20 datasets comparing memory performance for negative vs. neutral stimuli (see Figures 2, 3), 8 (*n* = 492; Wagner et al., 2007; Nishida et al., 2009; Prehn-Kristensen et al., 2009, 2011; Cunningham et al., 2014a; Morgenthaler et al., 2014; Göder et al., 2015; Bolinger et al., 2018) used intentional encoding, whereas 12 [*n* = 1,032; Sterpenich et al., 2007; Payne et al., 2008, 2015; Payne and Kensinger, 2011; Baran et al., 2012; Cunningham et al., 2014b; Bennion et al., 2015; Cellini et al., 2016; Jones et al., 2016 [Dataset 1], 2018; (Mantua et al., 2017; Tempesta et al., 2017)] used incidental encoding. Subgroup analyses detected no significant effect for either the sleep condition, *p* = 0.087, or for the waking control condition, *p* = 0.361.

Because only 1 study used intentional encoding when comparing positive to neutral memory performance (Wagner et al., 2007), we undertook no subgroup analysis in that regard. However, sensitivity analyses indicated that including or excluding the sleep deprivation study from this overall pool of datasets made little difference to the observed effect (see Table 2).

Only 1 study (Bennion et al., 2016) used intentional encoding and then compared combined negative and positive to neutral memory performance. Hence, we undertook no subgroup analysis in this regard. Sensitivity analyses indicated that the magnitude of the difference in effect size estimates for the two comparisons becomes larger when excluding the dataset reporting intentional encoding study (see Table 2). Although this finding suggests that the preferential consolidation of emotional over neutral material is observed more strongly after incidental rather than intentional encoding of information, this sensitivity analysis is identical to the one performed for the timing and duration of sleep. Therefore, it is impossible to determine which of these moderator variables (timing and duration of sleep condition, or type of encoding) drives this effect.

#### Type of Outcome Measure

This set of analyses indicated that sleep-dependent preferential consolidation of emotional over neutral material was more likely to be demonstrated in studies using free recall than recognition outcome measures.

While no studies compared free recall outcome measures for negative vs. neutral material, only 1 study (Chambers and Payne, 2014) reported these outcomes for a comparison of positive vs. neutral information, after either a sleep- or wake-filled delay. Hence, we undertook no subgroup analysis in this regard. However, sensitivity analyses for positive-neutral comparison indicated that including or excluding the dataset from the pool of studies made little difference to the observed effect (see Table 2).

Of the 4 datasets comparing memory performance for combined negative and positive vs. neutral stimuli after a sleep-filled delay (see Figures 6, 7), two (*n* = 269; (Schoch et al., 2017) [Dataset 1 and Dataset 2]) used a free recall outcome measure, *d* = 1.79, 95% CI [1.39, 2.19]. The other two (*n* = 152) made the performance comparison using a recognition outcome measure, *d* = 0.85, 95% CI [0.38, 1.32]. Subgroup analysis detected a significant difference, *p* = 0.003. This result suggests that, although emotional material is remembered significantly more readily than neutral material after a sleep-filled delay, this effect is particularly pronounced when participants are asked to recall the material spontaneously, without re-exposure to the stimulus.

When a similar subgroup analysis was applied to data from waking conditions, it detected no significant difference, *p* = 0.495. Taken together, these results suggest that the probability of observing preferential sleep-dependent consolidation of emotional over neutral material is higher in studies using free recall outcome measures than in those using recognition measures. This interpretation is tempered somewhat by the finding that the confidence intervals are wide and do overlap to some extent.

#### Gender Distribution of Sample

Of the 21 datasets comparing memory performance for negative vs. neutral stimuli after a sleep-filled delay (see Figure 2), two datasets [*n* = 128; (Prehn-Kristensen et al., 2009, 2011)] recruited only male participants, *d* = 1.08, 95% CI [0.55, 1.60]. The other 18 (*n* = 1,396) made the comparison using a mixed sample of male and female participants, *d* = 0.50, 95% CI [0.34, 0.65]. Subgroup analysis detected a significant effect, *p* = 0.039 suggesting that although both men and women remember negative material more readily than neutral material after a period of sleep, the effect is more pronounced in studies using male-only samples.

Of note here is that when a similar subgroup analysis was applied to data from waking conditions, it detected no significant difference, *p* = 0.709. These findings suggest that the probability of observing preferential sleep-dependent consolidation of emotional over neutral material is higher in all-male than in mixed-genders samples.

All datasets comparing memory performance for positive vs. neutral material, and combined negative and positive vs. neutral material, used mixed-gender samples.

#### Age Range of Sample

Among the set of 20 datasets comparing memory performance for negative vs. neutral material after either a sleep- or wake filled delay (see Figures 2, 3), 1 (*n* = 84; Jones et al., 2016 [Dataset 1]) used a sample of older adults aged >50 years, 3 (n = 192; Prehn-Kristensen et al., 2009, 2011; Bolinger et al., 2018) used samples of children aged < 18 years, and the remaining 16 (*n* = 1,248) used samples of young/middle aged adults aged 18-50 years. Analyses detected no significant subgroup effect after either a sleep- or a wake-filled delay, *p* = 0.522 and 0.926, respectively.

Only 1 study (Jones et al., 2016 [Dataset 2]) used a sample of older adults in comparing memory performance for positive vs. neutral material after either a sleep- or wake-filled delay. All other datasets making that comparison used samples of young/middle-aged adults. A sensitivity analysis indicated that removing this older-adult dataset from the overall pool resulted in a larger effect size difference between the sleep and the waking condition (see Table 2). This result suggests that, for the comparison of these types of material, sleep-dependent preferential consolidation of emotional over neutral material may be present for younger adults (18-45 years) but not older adults (>45 years).

All datasets comparing memory performance for combined negative and positive vs. neutral material used samples of adults.

#### Baseline Learning Control for Memory Outcome Measures

This set of analyses indicated that sleep-dependent preferential consolidation of emotional over neutral material was more likely to be demonstrated in studies that used baseline-controlled outcome measures (i.e., that used, as a primary outcome, a measure of retention, calculated as the score at delayed retrieval minus the score at pre-delay encoding) than in those studies that did not (i.e., that used post-delay recall or recognition scores as their outcomes).

Nine datasets recorded retention memory outcome measures (i.e. baseline-corrected measures) rather than post-delay means only. Statistically, it is not possible to include these retention values alongside uncontrolled post-delay mean scores in the same meta-analysis reporting standardized mean differences (Higgins and Green, 2011). Hence, we report here on separate analyses of datasets using retention scores. Given the small number of datasets, we did not undertake further subgroup or sensitivity analyses of the afore-described potential moderator variables.

**Figure 9:**
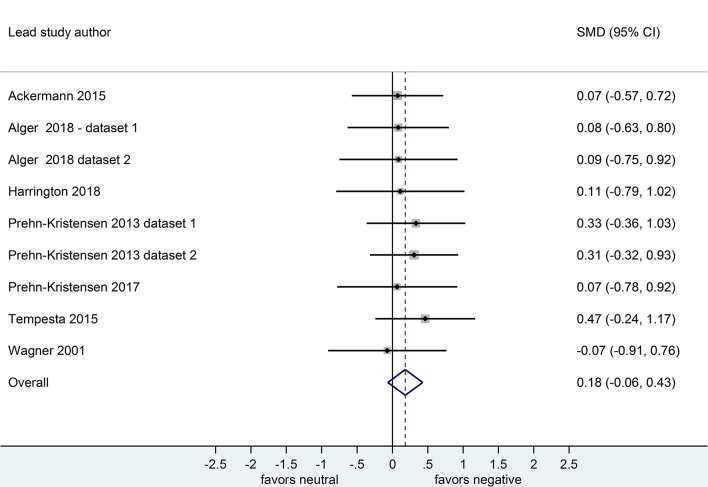
Memory performance for negative vs. neutral information after a wake-filled delay in datasets using baseline-controlled memory outcome measures (*k* = 9, *n* = 280). The *x*-axis represents effect size (Cohen's *d*). The dashed red line indicates the overall effect size, *d* = 0.18. Heterogeneity is reported as *I*^2^ = 0%, *p*_Q_ = 0.99. SMD, standardized mean difference; CI, confidence interval.

Nine datasets compared retention for negative vs. neutral stimuli after a sleep-filled delay (*n* = 297), *d* = 0.64, 95% CI [0.22, 1.07] or after a wake-filled delay (*n* = 280), *d* = 0.18, 95% CI [-0.06, 0.43]. This pattern of results suggests that sleep-dependent enhancement of emotional over neutral memory is observed clearly in studies reporting retention memory outcome measures (see Figures 8, 9).

**Figure 10:**
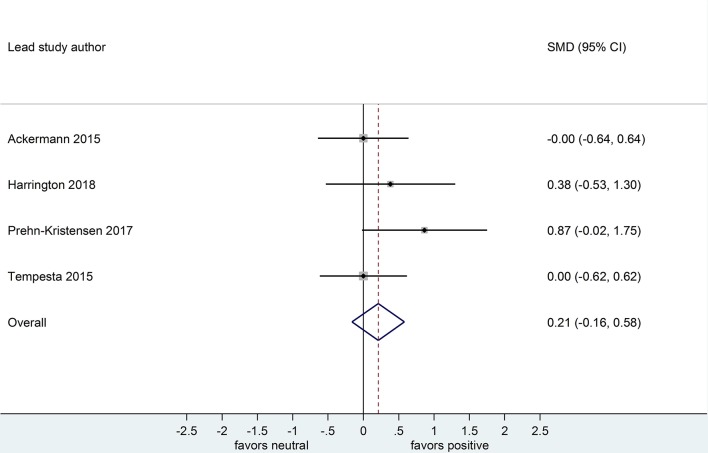
Memory performance for positive vs. neutral information after a sleep-filled delay in datasets using retention memory outcome measures (*k* = 4, *n* = 147). The *x*-axis represents effect size (Cohen's *d*). The dashed red line indicates the overall effect size, *d* = 0.21. Heterogeneity is reported as *I*^2^ = 3.2%, *p*_Q_ = 0.62. SMD, standardized mean difference; CI, confidence interval.

**Figure 11:**
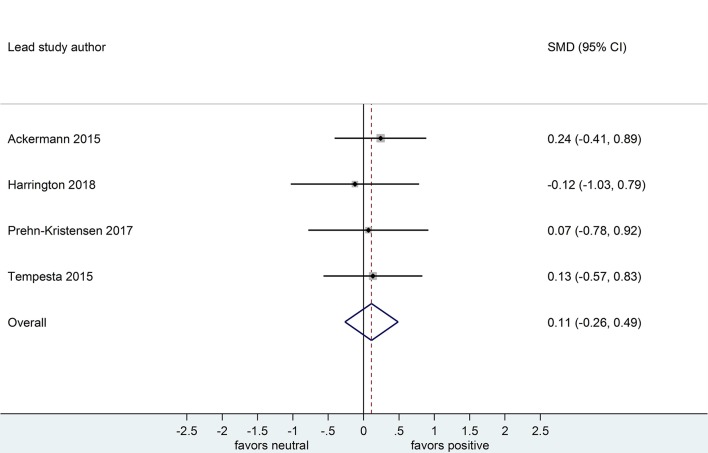
Memory performance for positive vs. neutral information after a wake-filled delay in datasets using retention memory outcome measures (*k* = 4, *n* = 134). The *x*-axis represents effect size (Cohen's *d*). The dashed red line indicates the overall effect size, *d* = 0.11. Heterogeneity is reported as *I*^2^ = 0 %, *p*_Q_ = 0.94. SMD, standardized mean difference; CI, confidence interval.

Four datasets compared retention for positive vs. neutral stimuli after a sleep-filled delay (*n* = 147), *d* = 0.21, 95% CI [-0.15, 0.58] or after a wake-filled delay (*n* = 134), *d* = 0.11, 95% CI [-0.26, 0.49]. Although the confidence intervals for these two analyses included a zero value and largely overlap, the difference between effect sizes is consistent with the pattern described for memory performance comparing negative vs. neutral material, albeit with a smaller sample of studies (see Figures 10, 11).

There were no datasets examining retention outcome measures in the combined negative and positive compared to neutral memory performance comparison.

#### Method of Data Extraction

For three datasets (*n* = 124; Atienza and Cantero, 2008; Payne et al., 2008; Bennion et al., 2016), all of which compared negative to neutral memory performance, we had to extract data using the WebPlotDigitizer program. Sensitivity analyses indicated that including or excluding this data from the overall pool of datasets made little difference to the observed effect (see Table 2).

## Discussion

Numerous studies suggest that whereas neutral memories tend to fade away over time, emotional memories are preferentially consolidated during sleep so that they remain stronger for longer (Wagner et al., 2001; Hu et al., 2006; Payne et al., 2008). However, not all studies replicate this result. The aim of this quantitative review was to evaluate the totality of evidence and to explore potential sources of inconsistency.

Our initial set of analyses assessed strength of memory for (i) valenced compared to neutral material after a sleep-filled interval, and (ii) valenced compared to neutral material after a wake-filled interval. The results showed that, after both sleep-filled and wake-filled delays, emotional material was remembered better than neutral material. Of primary importance here, then, was that there was no sleep-specific effect on memory for emotionally valenced (negative, positive, or combined negative-positive) material over neutral material.

Despite this negative result, further exploration of potential moderators suggested that two methodological conditions, in particular, might provide a better context for exploring whether sleep might provide a neurobiological environment that preferentially consolidates emotional over neutral material more strongly than waking does.

The first of these conditions involves the type of outcome measure used. We found that, in studies reporting free recall outcome measures, the magnitude of difference between strength of memory for emotional (specifically, combined negative-positive stimuli) vs. neutral material after a sleep-filled delay was significantly larger than that in studies reporting recognition measures. This subgroup difference was not significant when participants experienced a wake-filled delay between encoding and retrieval.

Few empirical studies in this literature make a direct comparison between memory performance on different outcome measures. Notably, however, Schoch et al. (2017) reported a pattern of data consistent with aggregate analyses within our review: They observed an enhancing influence of sleep on emotional memory when participants were asked to report images they remembered, but not when they were asked to complete a forced-choice recognition task. Moreover, a sizable literature suggests that two key memory processes under consideration here (retrieval during free recall tasks and consolidation during sleep) are reliant on hippocampal structure and activity (Girardeau et al., 2017; Miyamoto et al., 2017). Recognition-based retrieval, in contrast, relies more heavily on frontal-subcortical circuitry (Squire and Dede, 2015). An important consideration here, however, is the fact that free recall tasks tend to be more cognitively demanding than cued recall or recognition tasks. Hence, variation in task difficulty may be a confounding factor when attempting to describe whether, and how, differences in task type (free recall vs. recognition) might influence the ways in preferential consolidation of valenced over neutral material might be captured in the laboratory.

A second methodological condition that our review suggests may be important to uncovering sleep-dependent enhancement of emotional information is whether the post-delay memory outcome measure factors in a pre-delay control for initial learning. Specifically, our analyses suggested that, in studies subtracting performance on a pre-delay recall or recognition trial from performance on post-delay memory testing (i.e., studies reporting performance on what is commonly referred to as a measure of retention), there was post-sleep evidence of preferential consolidation of emotional over neutral material. In studies that did not control for initial learning, there was no evidence of this enhancing effect. This finding is consistent with the understanding that, in sleep research, baseline-controlled memory measures (i.e., of the amount of information retained after the delay, often reported as retention) are the best behavioral representations of memory consolidation (Antony and Paller, 2018).

Of note here is that this finding held in the relatively large group of studies (*k* = 6) comparing memory performance of negative vs. neutral material. Only two studies compared memory performance of positive vs. neutral material; no study compared memory performance of combined negative and positive vs. neutral material.

There are at least two plausible interpretations of this finding. One is that for sleep-dependent consolidation to occur, new learning may need to be actively brought to mind (i.e., reactivated) to ‘tag' it as relevant for consolidation during sleep (Antony and Paller, 2018). A second, and perhaps more prosaic, interpretation is that statistical differences, which are obscured by noise in the data, only become apparent after controlling for initial learning (for example, individual differences in learning capacity may obscure between-condition differences in memory consolidation). One way to test the strength of these two interpretations is to examine whether the exact same material recalled after initial learning is recalled again after the sleep- or wake-filled delay. This test is easy to apply in studies using free recall memory outcomes. However, recognition studies often use different subsets of the initial pool of learned material at immediate and post-delay recognition, and so in that context it is impossible to tease apart the merit of each interpretation.

Analyses of these two moderating variables (free recall vs. recognition as the type of outcome measure, and baseline-controlled or not as a feature of the memory measure) provide perhaps the most persuasive (and relevant, from the perspective of the sleep-memory literature) results from our secondary analyses. Analyses of the other moderating variables add methodological guidance for future research in this field.

First, our analyses suggested that future studies should carefully consider whether to use a nap or full-night paradigm. Specifically, excluding a nap study from the pool of studies comparing memory performance for combined negative-positive material to neutral material resulted in a larger post-sleep emotional-neutral memory difference. Two notes of caution here are that (a) this finding was only demonstrated for one of the three memory comparison conditions, and (b) the same dataset (Bennion et al., 2016) was removed as part of the sensitivity analyses for another moderator variable (type of memory encoding; it was the only study within this pool that used intentional encoding), and so we cannot be certain whether it was exclusion of the nap or exclusion of the intentional encoding that drove the change in effect. Given that only one of these accounts (the nap) drives the effect in the predicted direction, we favor this interpretation.

Second, studies that used sleep deprivation as a control for night-time sleep rather than regular day-time waking, showed a more pronounced emotional memory effect. That is participants experiencing sleep deprivation tended to remember both negative and positive information more readily than neutral information. This result is consistent with literature that demonstrates that sleep deprivation results in increased emotional reactivity both in reaction to negative (Yoo et al., 2007; van der Helm and Walker, 2011) and positive (Gujar et al., 2011; Krause et al., 2017) stimuli. Stronger emotional responses may result in stronger emotional memory for this category of stimuli. Methodologically studies should consider which kind of control condition they choose, because using the sleep deprivation paradigm may paradoxically result in participants remembering more emotional information after sleep deprivation rather than after sleep.

Third, our analyses revealed that studies that compared memory outcomes for combined negative-positive information with neutral information showed a stronger emotional memory effect than studies that compared a single valanced category to the neutral category. That is, the difference in memory strength for emotional material vs. neutral material was much larger in studies that combined positive and negative information into a single emotional category. Research findings consistently show that the strength of emotional response at encoding is highly correlated with the strength of memory recall or recognition after a delay (Sharot et al., 2004; Phelps, 2006). Studies that presented participants with both negative and positive emotional material may have achieved higher levels of emotion elicitation, whereas studies that presented either negative or positive information only may have dampened the strength of participants emotional experiences through desensitization (i.e. through repetitive presentation of material of the same valence; van den Hout et al., 2014; Ratneswaran et al., 2016).

Finally, our analyses indicated particular effects of sociodemographic variables on sleep-dependent consolidation of emotional memory. Specifically, both sample gender distribution (i.e., whether it was all male or mixed) and age (i.e., whether children, adults or older adults were studied) appeared to affect the size of effects.

Regarding gender, several previous studies (e.g., Debarnot et al., 2013; McDevitt et al., 2014; Sattari et al., 2017) have reported that, in women, sleep-dependent memory consolidation is affected by variations in female sex hormones (i.e., at some points in the menstrual cycle, effects are weaker than at others). Hence, studies recruiting male-only samples are more likely to observe consistent effects. However, no published study has specifically examined gender differences, or effects of menstrual cycle phase, on sleep-dependent consolidation of emotional material.

Regarding age, there are well-documented age-related differences in sleep architecture, memory processing, and sleep-dependent memory consolidation. Relative to young adults, older adults sleep more poorly, process learned material differently, and do not consolidate that material as effectively (Gui et al., 2017). Sensitivity analyses for positive vs. neutral material generated results consistent with this pattern: When the study containing an older-adult sample was removed, we observed preferential sleep-dependent consolidation of positive over neutral material. We did not, however, observe the same age-related effect within our largest subgroup analysis (for negative vs. neutral material). This inconsistency may be attributed to (a) the relatively small pool of studies we examined, and/or (b) the fact that we defined older adults here as those aged >45 years (rather than the more conventional >65 years).

## Limitations

The strength of our findings must be weighed against the following limitations. Primarily, as with any systematic review, the quality of our findings are constrained by the quality of the data we analyzed. Although we regard the included studies as being of sound design and without high risk of bias, there was substantial variability in their methodology. Hence, we had to conduct several subgroup and sensitivity analyses, and in most cases those analyses featured small numbers of studies and data derived from relatively low numbers of participants. Together, these factors constrain our ability to make strong inferences based on our moderator analyses.

Furthermore, we were unable to perform a direct statistical comparison of the sleep and waking conditions in addition to our primary comparison of memory for emotional vs. neutral stimuli. To compute the sleep-waking comparison, we would first need to calculate the difference between emotional and neutral memory for each condition separately and then compare those values meta-analytically. Although the difference of the means for emotional and neutral memory performance can be calculated reliably, the difference of the standard deviations (where standard deviations are a requirement of meta-analysis) cannot be computed in the same way.

We also note that although our analyses investigated quantitative influences on emotional memory, we did not explore whether sleep might induce qualitative/transformational changes in emotional memories. For example, a large body of work has demonstrated that sleep induces an “emotional trade-off,” wherein core emotional aspects of a memory episode are retained at the cost of forgetting non-emotional details (Payne et al., 2008, 2015; Cunningham et al., 2014a,b; Alger et al., 2018).

## Summary and Conclusion

Our first major finding was that the extant literature does not conclusively demonstrate that sleep preferentially consolidates emotional over neutral memories. However, our secondary analyses demonstrated that specific methodological features allowed such preferential consolidation to be observed. Most persuasive are the analyses suggesting that studies using free recall rather than recognition outcome measures, and baseline-controlled rather than uncontrolled post-sleep outcome measures, are more likely to demonstrate the effect.

Much attention has been paid to research investigating the functional value of sleep for cognitive processes, and especially for various forms of memory. There is widespread agreement that sleep plays a vital role in consolidating previously-learned information, and that the associated emotional valence may play an organizing role in how that information is processed during sleep. Neuroscientists will acknowledge, however, that there is much work to be done on the mechanisms underlying sleep-dependent emotional memory consolidation. We hope the current findings, and especially our proposition that particular aspects of study design (e.g., the types of sleep and waking control conditions, the categories of stimuli to which participants are exposed, and the outcome measures used) are critical, will guide future research in this field.

## Author Contributions

GL and EB conceptualized the meta-analysis and first-screened the papers. GL, EB, KT, and DB second-screened the papers. GL, EB, and KT performed data extraction. BS conducted the data analysis and all authors were involved in manuscript preparation.

### Conflict of Interest Statement

DB would nevertheless like to declare that he is Chair of the Psychopharmacology Committee of the Royal College of Psychiatrists, Clinical Advisor to the National Clinical Audit of Anxiety and Depression, and a Medical Patron of Anxiety UK. The remaining authors declare that the research was conducted in the absence of any commercial or financial relationships that could be construed as a potential conflict of interest.
